# Ocular features in Aicardi syndrome: A case report

**DOI:** 10.1097/MD.0000000000031950

**Published:** 2022-12-09

**Authors:** Sebastian Sirek, Erita Filipek, Bogumiła Wójcik–Niklewska, Dorota Pojda-Wilczek, Ewa Mrukwa-Kominek

**Affiliations:** a Department of Ophthalmology, Faculty of Medical Sciences in Katowice, Medical University of Silesia in Katowice, Katowice, Poland; b Kornel Gibiński University Clinical Centre, Katowice, Poland; c Department of Pediatric Ophtalmology, Faculty of Medical Sciences in Katowice, Medical University of Silesia in Katowice, Katowice, Poland.

**Keywords:** Aicardi syndrome, chorioretinal lacunae, coloboma, VEP

## Abstract

Aicardi syndrome is a genetic malformation syndrome with a triad of dysgenesis or agenesis of the corpus callosum, distinctive chorioretinal lacunae and infantile spasms. It is a rare developmental disorder first described in 1965. The disease affects 1 in 100,000 live births.

**Patient concerns:**

We describe a 34-month-old girl diagnosed with Aicardi Syndrome.

**Diagnosis:**

Based on the results of color images of the fundus, medical history as well as the analysis of karyotype and DNA microarrays, the patient was diagnosed with Aicardi’s syndrome.

**Interventions:**

Additionally an B-scan ultrasonography and an electrophysiological test was performed.

**Outcome:**

Fundoscopic examination revealed optic disc colobomas in both eyes, extensive chorioretinal lacunae at the posterior pole with retinal pigment epithelium regrouping and atrophy. Flash visual evoked potentials (FVEP) P2 amplitude was lower than normal range. B-scan ultrasonography revealed an optic disc lesion consistent with optic disk coloboma.

**Lessons:**

Children with congenital central nervous system malformations should undergo regular ophthalmic checkups to facilitate diagnosis and determine prognosis of visual function development.

## 1. Introduction

Aicardi syndrome is a genetic malformation syndrome with a triad of dysgenesis or agenesis of the corpus callosum, distinctive chorioretinal lacunae and infantile spasms. It is a rare developmental disorder first described in 1965 by Jean Aicardi, a French neurologist. The disease affects 1 in 100,000 live births. The diagnosis of Aicardi syndrome is based exclusively on clinical findings. The presence of the classic triad is diagnostic for Aicardi syndrome. The presence of 2 of the classic triad plus at least 2 other major or supporting features is strongly suggestive of the diagnosis of Aicardi syndrome.^[1–9]^ The classical triad includes: agenesis of the corpus callosum, distinctive chorioretinal lacunae, infantile spasms. The major features include: cortical malformations (mostly polymicrogyria), periventricular and subcortical heterotopia, cysts around third cerebral ventricle and/or choroid lexus, optic disc/nerve coloboma or hypoplasia. The supporting features include: vertebral and rib abnormalities, microphthalmia, “split-brain” electroencephalogram, gross cerebral hemispheric asymmetry, vascular malformations or vascular malignancy. The aim of this report is to present a child with ocular features characteristic of Aicardi syndrome hospitalized for observation widening, extensive diagnostics in the Pediatric Ophthalmology Department of Kornel Gibiński University Clinical Centre in Katowice.

## 2. Case report

**Figure 1.**
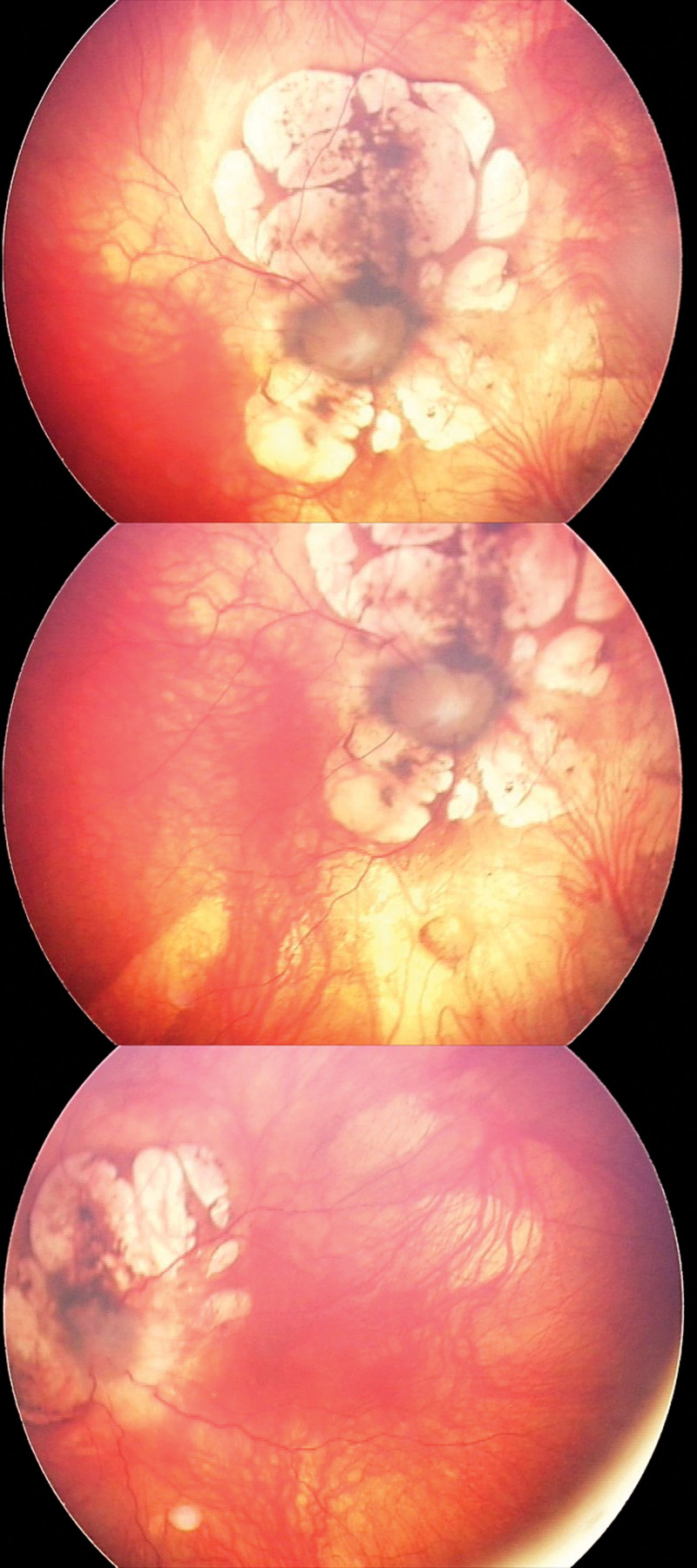
Color fundus images.

**Figure 2.**
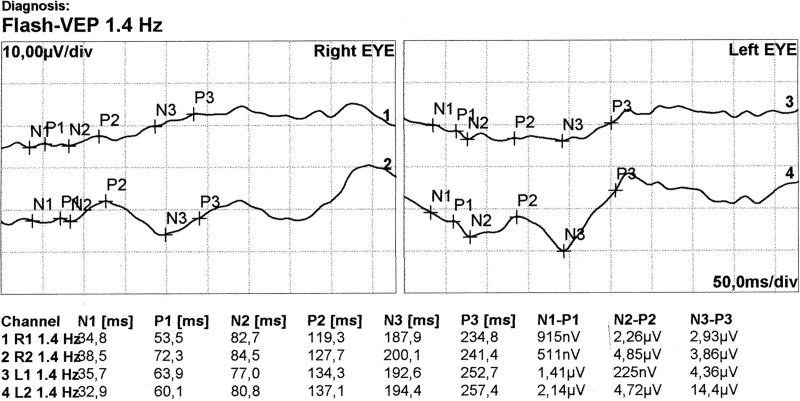
The result of electrophysiological test—flash VEP. Channels R1, L1: O_1_, left brain hemisphere; Channels R2, L2: O_2_, right brain hemisphere. FVEP = flash visual evoked potentials.

**Figure 3.**
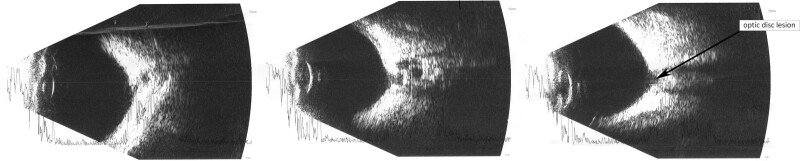
The result of B-scan ultrasonography examination.

A 34-month-old girl born in the 42 weeks of pregnancy with congenital abnormalities of the central nervous system such as: holoprosencephaly, schizencephaly, polymicrogyria, agenesis of corpus callosum was examined in the Pediatric Ophthalmology Department of Kornel Gibiński University Clinical Centre in Katowice There were also observed epilepsy and ocular fundus lesions. The Genetics Clinic at the University Children’s Hospital of Cracow performed an analysis of the patient’s karyotype, DNA microarray and a genetics database (London Medical Databases). Ophthalmic examination was performed under general anesthesia. Additionally an B-scan ultrasonography and an electrophysiological test was performed. Flash visual evoked potentials (FVEP) were tested, using the Reti-Port electrophysiological device from Roland Consult (Germany), in accordance with the standards of the International Society for Clinical Electrophysiology of Vision.^[10]^ Skin gold-cup electrodes (active electrodes were placed at O_1_ and O_2_, reference one at Fz) and standard flashes with a frequency of 1.4 Hz were used in the Ganzfeld stimulator. Amplitude and latency of P2 wave were measured. The reference interval ranged from 2.5-97.5%. Color fundus images were obtained with RetCam 3 (Clarity Medical Systems, Inc., Pleasanton, CA). No pathology of the anterior segment of the eye was revealed. The patient exhibited bilateral pupillary response to light; the left pupil response was sluggish. Intraocular pressure was 10 mm Hg in both eyes; central retinal thickness was 613 µm and 588 µm in the right and left eye, respectively. Gonioscopy grading: wide angle with normal structure with single iris bridges was demonstrated (C30 r0 PTM). Fundoscopic examination revealed optic disc colobomas in both eyes, extensive chorioretinal lacunae at the posterior pole with retinal pigment epithelium regrouping, peripheral retinal pigment epithelium atrophy and retinal vessel narrowing (Fig. 1). The electrophysiological test showed that FVEP P2 wave latency was normal (119–137 ms; normal values 96–148 ms; mean 114 ms; median 113.5 ms). The P2 amplitude after stimulation of right eye and left eye recorded from the O_1_ was 2.26 µV and 225 nV and from the O_2_ was 4.85 and 4.75 µV, respectively, (normal values: 5.9–39.1 µV; mean 14.9; median 12.7 µV) (Fig. 2). Low P2 amplitude might be indicative of optic nerve hypoplasia. These are the first clinical data recorded on FVEP recording in Aicardi Syndrome. B-scan ultrasonography revealed an optic disc lesion consistent with optic disk coloboma (Fig. 3). A normal female karyotype 46,XX was found (GTG-banding) indicating no chromosomal rearrangement. DNA microarray was also performed to extend the diagnostic process. Since no chromosome abnormalities were detected, deletions and duplications of more than 200,000 base pairs were excluded as the cause of clinical symptoms.

## 3. Discussion

Aicardi syndrome develops due to a mutation in a gene located on the X chromosome (locus Xp22). The inheritance is X-linked dominant; the mutation can occur in both females and males but in males it is lethal early in embryonic development. The gene responsible for Aicardi syndrome has not been identified. The disease affects 1 in 100,000 live births and primarily occurs in girls.^[1–6]^ Most patients die at an early age, most frequently due to aspiration pneumonia but some survive into adolescence or adulthood.^[8]^ An analysis of a genetics database (London Medical Databases) indicates Aicardi syndrome might underlie the clinical presentation seen in our patient. However, considering the unclear etiology of the disorder, this is a clinical diagnosis and cannot be confirmed by molecular genotyping. Apart from chorioretinal lacunae, patients with Aicardi syndrome have been diagnosed with optic nerve coloboma or atrophy which were also seen in our patient. Other ophthalmic findings include microphthalmia, cataract, iris synechiae, optic disc pigmentation, retrobulbar cysts, retinal detachment or macular scars.^[9]^ While there is no known cure for Aicardi syndrome, there are treatments that can help control symptoms. Pharmacotherapy treatment for associated diseases in Aicardi syndrome included corticotropin, prednisone, valproic acid and clonazepam but with variable success. Other treatment may include physical therapy, speech therapy, and occupational therapy, as well as support for skeletal and muscle problems to prevent scoliosis related complication. Speech is usually very limited, while other abilities and disabilities vary greatly. When the macula and optic nerve are not affected, the prognosis for development of visual function is favorable.^[1–6]^

## 4. Conclusions

Children with congenital central nervous system malformations should undergo regular ophthalmic checkups to facilitate diagnosis and determine prognosis of visual function development. Detailed genetic analysis and clinical evaluation allow to determine the diagnosis.

## Acknowledgments

The authors are grateful to the patient and her family for their support of this article. The authors would like to thank Magdalena Janeczko for prepring an analysis of the patient’s karyotype, DNA microarray and a genetics database.

## Author contributions

All authors contributed to the study conception and design. All authors participated in the study, diagnosis and treatment of the patient described in the article. Material preparation, data collection and analysis were performed by Sebastian Sirek and Bogumiła Wójcik - Niklewska. The draft of the manuscript was written by Sebastian Sirek. All authors commented on previous versions of the manuscript. All authors read and approved the final manuscript.

**Conceptualization:** Sebastian Sirek, Erita Filipek, Bogumiła Wójcik–Niklewska, Dorota Pojda-Wilczek, Ewa Mrukwa-Kominek.

**Data curation:** Sebastian Sirek, Bogumiła Wójcik–Niklewska, Dorota Pojda-Wilczek.

**Formal analysis:** Sebastian Sirek, Erita Filipek, Bogumiła Wójcik–Niklewska, Dorota Pojda-Wilczek.

**Funding acquisition:** Sebastian Sirek.

**Investigation:** Sebastian Sirek, Bogumiła Wójcik–Niklewska.

**Methodology:** Sebastian Sirek.

**Project administration:** Sebastian Sirek, Erita Filipek, Dorota Pojda-Wilczek, Ewa Mrukwa-Kominek.

**Resources:** Sebastian Sirek.

**Software:** Sebastian Sirek.

**Supervision:** Sebastian Sirek, Erita Filipek, Dorota Pojda-Wilczek, Ewa Mrukwa-Kominek.

**Validation:** Sebastian Sirek, Erita Filipek, Dorota Pojda-Wilczek.

**Visualization:** Sebastian Sirek.

**Writing – original draft:** Sebastian Sirek.

**Writing – review & editing:** Sebastian Sirek,
